# Estrogen-Related Receptor Alpha: An Under-Appreciated Potential Target for the Treatment of Metabolic Diseases

**DOI:** 10.3390/ijms21051645

**Published:** 2020-02-28

**Authors:** Madhulika Tripathi, Paul Michael Yen, Brijesh Kumar Singh

**Affiliations:** Laboratory of Hormonal Regulation, Cardiovascular and Metabolic Disorders Program, Duke-NUS Medical School, Singapore 169857, Singapore; madhulika.tripathi@duke-nus.edu.sg (M.T.); paul.yen@duke-nus.edu.sg (P.M.Y.)

**Keywords:** estrogen-related receptor alpha, mitophagy, mitochondrial turnover, metabolic diseases, non-alcoholic fatty liver disease (NAFLD), adipogenesis, adaptive thermogenesis

## Abstract

The estrogen-related receptor alpha (ESRRA) is an orphan nuclear receptor (NR) that significantly influences cellular metabolism. ESRRA is predominantly expressed in metabolically-active tissues and regulates the transcription of metabolic genes, including those involved in mitochondrial turnover and autophagy. Although ESRRA activity is well-characterized in several types of cancer, recent reports suggest that it also has an important role in metabolic diseases. This minireview focuses on the regulation of cellular metabolism and function by ESRRA and its potential as a target for the treatment of metabolic disorders.

## 1. Introduction

When the estrogen-related receptor alpha (ESRRA) was first cloned, it was found to be a nuclear receptor (NR) that had DNA sequence homology to the estrogen receptor alpha (ESR1) [1]. There are several examples of estrogen-related receptor (ESRR) and estrogen-signaling cross-talk via mutual transcriptional regulation or reciprocal binding to each other’s response elements of common target genes in a context-specific manner [2,3]. However, subsequent ligand binding and reporter-gene transfection studies surprisingly demonstrated that estrogens did not bind to ESRRs with a high affinity, and did not directly regulate their expression and/or transcriptional activity. Thus far, with the possible exception of cholesterol, there have not been any endogenous ligands identified for ESRRA; hence, it is still considered an “orphan” nuclear receptor that does not have a *bona fide* endogenous ligand [1,4,5]. ESRRs belong to a small subfamily of nuclear receptors called NR3B and consisting of three members: estrogen-related receptor alpha (ESRRA/NR3B1), beta (ESRRB/NR3B2), and gamma (ESRRG/NR3B3) [6,7,8,9]. Among them, ESRRA and ESRRG are mostly expressed in metabolically-active tissues that preferentially utilize fatty acids as fuel (e.g., heart, brown adipose tissue (BAT), cerebellum, intestine, and liver) [1,7,10]. ESRRA regulates mitochondrial activity, biogenesis, and turnover, as well as lipid catabolism [11,12,13,14,15]. ESRRA also regulate normal physiological and developmental functions in muscles and bone [16,17]. Surprisingly, although ESRRA regulates the mitochondrial function and lipid catabolism, ESRRA-null mice have displayed a general decrease in fat mass and resistance to high-fat diet-induced obesity [18]. This phenotype is most likely due to reduced adipogenesis, as well as the down-regulation of lipid metabolism and adipogenic pathways that are regulated by other NRs [18,19,20]. However, ESRRs also play a key role in the adaptive thermogenesis of BAT during adrenergic stimulation, and the loss of all ESRR isoforms in adipose tissue significantly decreases the metabolic benefits of adrenergic agonism during obesity [21,22]. Similarly, increasing the expression of peroxisome proliferator-activated receptor gamma coactivator 1 alpha (PPARGC1A), the protein ligand of ESRRA, increases energy expenditure and reduces obesity [23]. So far, most previous studies of ESRR actions have focused on its role in cancer progression and this topic has been reviewed elsewhere [24,25,26,27,28,29,30,31,32,33,34,35]. There have also been a few studies on its roles in bone and muscle differentiation [36,37,38], trauma/shock [39], and infection [40]. The physiological and metabolic roles of ESRRA in vivo have only been appreciated recently and are still not fully understood. This review will focus on the role of ESRRA in metabolism and metabolic disorders.

## 2. Regulation of ESRRA

### 2.1. ESRRA Interaction with Co-Regulators to Regulate Its Own Expression

In the absence of a ligand, the ESRR ligand binding domain (LBD) is in an active conformation [1,41] and is constitutively active. Endogenous small molecule ligands for ESRR have yet to be identified, so it is still categorized as an orphan NR [1]. However, PPARGC1A, serves as a *bona fide* endogenous “protein ligand” and co-activator for ESRRA [14,42]. PPARGC1A is recruited by NRs to NR response elements commonly located in the promoters of target genes to increase gene expression [14,42]. Similar to other NRs that bind ligands, the carboxy-terminal ligand binding domain (LBD) of ESRRA interacts with PPARGC1A. ESRRA has three protein interaction sites (LxxLL) in the LBD, two of which act as NR interaction sites. However, the third ESRR protein interaction site contains an atypical LLxYL motif that specifically permits ESRR to interact with, and activate, other ESRRs [14,41,43,44]. When PPARGC1A expression is low, ESRRA has weak transcriptional activation; however, when PPARGC1A is overexpressed in transformed cells, ESRRA becomes a potent transcriptional activator [14,23,41,43,44]. Additionally, PPRAGC1A regulates ESRRA expression in a feed-forward loop as the ESRRA/PPARGC1A dimer binds to conserved ESRRA response elements (ERREs; TNAAGGTCA) in the *ESRRA* gene promoter region [45,46]. With regards to this connection, *ESRRA* mRNA expression is also high in tissues that have high PPARGC1A/B expression. The formation of a PPARGC1A/B-ESRRA dimer may help stabilize ESRRA binding to the promoter and stimulate ESRRA transcriptional activity.

Unlike PPARGC1A, which is a positive regulator of ESRRA activity, nuclear receptor co-repressor 1 (NCOR1; also known as thyroid hormone (TH) and retinoic acid receptor-associated co-repressor 1 (TRAC-1)) exerts opposing effects on the transcriptional activity of ESRRA [47,48]. NCOR1 is a basal co-repressor that recruits histone deacetylases (HDACs) to the promoter region in the absence of a ligand. Its binding to the ESRRA LBD leads to the repression of ESRRA-mediated transcriptional activity during certain physiological conditions, such as the fed state [47,49]. Moreover, ESRRA transcriptional activity is also repressed by its interaction with receptor interacting protein 140 (RIP140) and PROX1 [50,51,52].

### 2.2. Identification of Potential ESRRA Ligands

Recent studies suggest that endogenous cholesterols may be potential ESRRA ligands [31,53]. Moreover, PPARGC1A increases cholesterol–ESRRA interaction and cholesterol stabilizes the ESRRA-PPARGC1A complex [53,54]. However, it still is controversial whether cholesterol or other related compounds are the natural endogenous ligands for ESRRA. Although synthetic antagonists have been developed, the identification and development of synthetic ESRR-specific agonists have been very challenging. Recent efforts to identify ESRRA ligands using small molecule libraries such as the Library of Pharmacologically Active Compounds (LOPAC) and Tox21 compound library have identified forskolin, phorbol 12-myristate 13-acetate (3MA), and several statins (atrovastatin, cerivastatin, fluvastatin, mevastatin, and lovastatin), as agonists for ESRRA or ESRRA/PPARGC1A dimers [55,56]. However, statins (inhibit the gene expression of HMG-CoA reductase, a key enzyme in the mevalonate pathway) used to reduce LDL-cholesterol and the intracellular cholesterol level, and thus oppose ESRRA activity in vivo [53]. Furthermore, statins increase hepatic gluconeogenesis by up-regulating the mRNA expression of *PCK1*, a gene that is negatively regulated by ESRRA [57,58,59]. Therefore, although statins may have agonist effects in vitro, their net effects in vivo may actually oppose those of ESRRA. Interestingly, genistein (a phytoestrogen) and several plant-derived compounds (apigenin, resveratrol, rutaecarpine, and flavone) are also agonists for ESRRA and the ESRRA-PPARGC1A dimer. Additionally, several pesticides (such as acriflavine, cholemidazole, pyridaben, and trifloridine), antineoplastic agents (such as artemisinine, bortezomib, etoposide, and Vorinostat) and other compounds, such as rotenone, camptothecin, thapsigargin, papverine, staurosponine, and progesterone, may be ESRRA antagonists, ESRRA/PPARGC1A antagonists, or both [56,60]. Taken together, these screening studies suggest that several ESRRA ligands have the potential to be therapeutic agents that enhance ESRRA activity. However, little is known about these compounds’ effects in the normal physiological state and in disease. These recent data also suggest that ESRRA should probably no longer be classified as an orphan NR, even though there is no consensus on its endogenous ligand.

### 2.3. Other Mechanisms for Regulating ESRRA Activity

ESRRA transcriptional activity can be co-regulated by other factors, as well as by post-transcriptional modifications. Chaveroux et al. showed that mammalian target of rapamycin (MTOR) and ESRRA both regulate common metabolic pathways, such as lipid homeostasis and the tricarboxylic acid cycle (TCA) cycle. Using ChIP-seq, they showed that MTOR and ESRRA share many target genes, and interact with each other in similar regulatory regions of these genes on a genome-wide scale [61]. Likewise, TH regulates ESRRA transcriptional activity by enhancing PPARGC1A expression and stimulating ESRRA expression [62]. Interestingly, ChIP-seq analysis also shows that there are a significant number of genes that contain both TH receptor beta (THRB) and ESRRA binding sites in the promoters of common target genes involved in mitochondrial oxidative phosphorylation (OXPHOS), the TCA cycle, etc. [62], raising the possibility that they co-regulate these genes.

The activity of ESRRA is also regulated by post-translational modifications. In particular, the protein expression of ESRRA is regulated by the ubiquitin-proteasome system. In Parkinson’s disease, parkin E3 ubiquitin ligase enhances ESRRA degradation and causes dopamine toxicity and oxidative stress [63]. Furthermore, MTOR also increases ESRRA protein stability by regulating *UBB* and *STUB1* genes [61]. ESRRA activity also is regulated by post-translational modifications such as phosphorylation (by EGF, MAPK, AKT, and cAMP) and theacetylation/deacetylation of ESRRA itself, and/or its co-activators, such as PPARGC1A and p300 (by PCAF, SIRT1, SIRT5, HDAC3, HDAC5, and HDAC8), as well as by microRNAs [1,64].

## 3. ESRRA in Metabolism

### 3.1. ESRRA Regulation of Oxidative Metabolism and Adaptive Energy Metabolism

Liver is the central tissue responsible for energy homeostasis during fasting and calorie restriction (CR). However, BAT is the critical regulator of adaptive energy metabolism during acute cold exposure. Fasting, CR, cold exposure, and exercise have all been shown to induce ESRRA expression and activity in specific tissues [1,2,65,66]. Transcriptome data from different tissues show that ESRRA-PPARGC1A regulates hundreds of genes involved in OXPHOS, the TCA cycle, fatty acid beta-oxidation (FAO), and glucose and lipid metabolism [1,14,20,21,22,27,65]. Moreover, ESRRA also regulates mitochondrial biogenesis, mitophagy, and mitochondrial turnover by directly inducing *TFB2M, TFAM, NRF1, MFN2,* and *SIRT3* gene expression [62,67]. The *TFB2M*, *MFN2,* and *SIRT3* promoters contain functional ESRR binding sites that activate transcription by recruiting the ESRRA-PPARGC1A complex [67,68]. In the liver, TH induces the expression of ESRRA, which then increases the expression of the autophagy-regulating kinase ULK1. This leads to increased mitophagy, as ULK1 is recruited to the mitochondria and activates FUNDC1 to promote DRP1-mediated mitochondrial fission that leads to mitochondrial interaction with the autophagosomal protein, MAP1LC3B-II [62].

Brown et al. [22] recently showed that both ESRRA and ESRRG are necessary for the adrenergic-stimulated transcriptional reprogramming of BAT; each has redundant effects on target gene transcription. In mice lacking both ESRRA and ESRRG isoforms, specifically in adipose tissue (adipose ESRR-DKO), there was a decreased oxidative and thermogenic capacity that caused them to rapidly become hypothermic when exposed to cold. When these mice were fed a high-fat diet, they did not display any weight change or show any improvement in glucose tolerance when treated with beta3-adrenergic agonists. Consistent with previous findings from other laboratories, they also showed that the ESRRA expression in BAT and muscle is upregulated during cold exposure and exercise [7,20,21,65,69]. Since there are increased mitochondria when there is a high expression of ESRRA, PPARGC1A, and PPARGC1B in BAT, ESRRA is thought to be critical for adaptive thermogenesis during cold stress. Interestingly, ESRRA null mice are cold intolerant and unable to maintain their core body temperature [21]. However, in contrast to the work of Brown et al., the defect in temperature control is not due to impaired beta-adrenergic signaling, but to intrinsic respiratory defects in the ESRRA null adipocytes. Furthermore, these ESRRA null adipocytes do not show any alteration in the expression of the thermogenic genes *UCP1* and *DIO1*, whereas adipose ESRR-DKO mice show a significantly lower expression of these genes, as well as the genes regulating the TCA cycle, OXPHOS, FAO, and mitochondrial biogenesis. These latter data support the notion that ESRRA and ESRRG are both required to co-regulate some genes involved in adaptive thermogenesis in BAT. On the other hand, a study on fetal mesenchymal stem cells that express brown fat-determining factor PRDM16 showed that UCP1 expression can be dependent upon ESRRA expression alone in some cell types [70].

Unlike its actions in BAT, ESRRA negatively regulates *UCP1* and *DIO1* gene expression and positively regulates differentiation and *FASN* expression in white adipose tissue (WAT) [18,20]. Similarly, ESRRA null mice are resistant to developing high fat-induced obesity without altering the energy expenditure [18]. Furthermore, PPARGC1A and PPARGC1B are important during ESRRA-mediated adipogenesis [19,20].

### 3.2. ESRRA Regulation of the Metabolic Clock

Energy metabolism and the circadian rhythm are closely related and interlinked metabolic processes. Disruption of the circadian rhythm can lead to energy imbalance and an increased risk for metabolic diseases [71,72]. Interestingly, the temporal oscillation of ESRRA expression is synchronized with circadian clock gene expression in many tissues [73,74,75,76,77]. ESRRA, as well as PPARGC1A and PPARGC1B, exhibit diurnal expression patterns in diverse tissues such as the liver, muscle, kidney, and bone, and play a role in regulating core clock genes [74,75,78]. In the liver, ESRRA is a critical regulator of diurnal glucose, triglycerides, cholesterol, bile acid, and insulin levels, as well as the expression of core-clock and clock control genes in the liver [79]. These circadian rhythm- associated actions of ESRRA seem to be co-regulated by core-clock regulators BAML1 and PROX1 [52,79]. Although the circadian regulation of ESRRA has been known for more than a decade, its precise role in the diurnal regulation of metabolism still needs further elucidation.

## 4. Therapeutic Relevance of ESRRA in Metabolic Diseases

Due to the complex nature of the metabolic actions of ESRRA and the co-regulation of many of its metabolic pathways by multiple cell signaling pathways and other transcription factors, it is difficult to understand all of ESRRA’s effects on cellular metabolism, especially in vivo. Current research has established that ESRRA plays a central role in hepatic and BAT energy metabolism during the normal physiological state, as well as in pathological conditions. Both tissues also crosstalk with other tissues to maintain metabolic homeostasis in response to environmental and physiological stresses, such as fasting, CR, exercise, etc. Therefore, disturbances in liver and BAT nutrient- and energy-sensing pathways, such as MTOR and AMPK, can lead to decreased ESRRA expression or activity and contribute to the development of metabolic disorders, such as obesity, non-alcoholic fatty liver disease (NAFLD), steatohepatitis (NASH), insulin resistance, and type 2 diabetes.

### 4.1. ESRRA as a Target for Obesity

Early studies showed that ESRRA stimulates adipogenesis and lipogenesis in vivo [19,20]. On the other hand, ESRRA also increases the energy expenditure and thermogenesis [21,22], so it is not easy to predict a priori what impact, if any, activating or inactivating ESRRA would have on obesity. When whole-body ESRRA knockout mice are fed a high-fat diet, they are resistant to diet-induced obesity because of increased lipid catabolism, reduced adipogenesis, and impaired fat absorption and transport through the gut [18,65,80]. In contrast, the ESRRA23 polymorphism is associated with a significantly higher BMI and may be a genetic factor contributing to human obesity [81]. When the phenotypes of high-fat diet-induced obese (DIO) mice were compared with high-fat diet-induced obesity-resistant (DIO-R) mice, the DIO-R mice had significantly higher levels of AMPK activation, as well as PPARGC1A and ESRRA expression [82]. Likewise, *Lepr-*deficient db/db mice (obese and diabetic mice phenotype) and mice fed a high-fat diet showed suppressed PPARGC1A and ESRRA expression, as well as reduced adipose mitochondrial ATP production and function [83]. Interestingly, rosiglitazone treatment restores adipose PPARGC1A and ESRRA expression and re-establishes normal adipose mitochondrial biogenesis and activity [83]. PPARGC1B transgenic mice, which overexpress PPARGC1B as an ESRRA ligand, exhibit an increased expression of the MCAD (a known ESRRA target gene) and display a phenotype similar to those found in athletes [23]. These mice are hyperphagic, have an elevated energy expenditure, and are resistant to high-fat diet-induced obesity [23]. Moreover, adipose-specific *FLCN* null mice exhibit increased energy expenditure and protection from high-fat diet (HFD)-induced obesity through the chronic hyperactivation of AMPK, showing activation of the PPARGC1A-ESRRA axis in adipose tissue. This leads to the upregulation of metabolic genes that promote mitochondrial biogenesis and activity [84]. Furthermore, genistein, a plant-derived compound, has recently been found to be an ESRRA agonist by small molecule screening [55,56,85], and improves diet-induced obesity by increasing energy expenditure, lipid catabolism via AMPK activation, and the serum TH concentration [86,87,88,89]. TH activates the PPARGC1A-ESRRA axis to increase mitochondrial biogenesis, activity, and turnover [62]. Although confirmatory studies need to be performed in ESSRA knockout mice, it is likely that ESRRA mediates many of genistein’s beneficial effects on diet-induced obesity.

### 4.2. ESRRA as a Target for NAFLD

Major sources of hepatic fat accumulation are from dietary fat, de novo lipogenesis, and lipolysis from WAT. The chronic accumulation of hepatic triglycerides and saturated fatty acids increases oxidative stress and inflammation that activate hepatic stellate cells. This leads to fibrosis in NASH that can advance to hepatic cirrhosis [90,91,92]. Given its important role in energy expenditure and mitochondrial activity, the activation of ESRRA and its downstream pathways could be therapeutically beneficial for NAFLD. Chaveroux et al. showed that the genetic and pharmacological inhibition of ESRRA activity exacerbated hepatosteatosis in rapamycin-treated mice [61]. In contrast, B’Chir et al. showed that the genetic ablation of ESRRA protected against fatty liver induced in DIO [93]. Consistent with the findings in ESRRA null mice, ESRRA inverse agonists, compound 29 (C29) and compound 50 (C50), also show obesity resistance in DIO mouse models [94,95,96]. Recent studies on the ESRRA activator, genistein, have shown beneficial effects in preclinical dietary NAFLD models, as well as in a randomized, controlled clinical trial on NAFLD patients [97,98,99,100,101]. Genistein increases AMPK activity, lipid catabolism, and mitochondrial activity, while reducing de novo lipogenesis. Genistein also induces many ESRRA target genes, such as *MCAD, HMGCS2, CPT1A, PPARA*, and *PPARGC1A*, suggesting that these beneficial effects of genistein on NAFLD may occur by virtue of its ability to be an ESRRA agonist [97,101]. While these results are promising, these studies need to be confirmed using liver-specific ESRRA knockout mouse models of NAFLD as a negative control for ESRRA-specific actions by genistein.

### 4.3. ESRRA as a Target for Insulin Resistance and Type 2 Diabetes

Insulin resistance is characterized by a reduced response to insulin actions at physiological, cellular, and molecular levels. It is a hallmark of type 2 diabetes and is a major contributor to the development of NAFLD and vice versa [102]. While obesity is associated with chronic inflammation that leads to insulin resistance and NAFLD [90], mitochondrial dysfunction is involved in all three metabolic disorders [102,103,104,105]. Most notably, mitochondrial defects in FAO lead to the accumulation of intracellular fatty acid metabolites such as dihydroxyglycerol and ceramides that adversely affect insulin signaling in various tissues [106,107,108]. Similarly, many of the genes that are regulated by transcription factors and NRs also play a critical role in the development of insulin resistance [109,110]. Earlier studies in diabetic patients show that OXPHOS genes are downregulated in muscle, but these genes can be upregulated in a PPARGC1A-ESRRA-dependent manner in C2C12 myotubes [43,111]. Furthermore, ESRRA-regulated genes are decreased in insulin-resistant patients, and there is a correlation between insulin sensitivity and ESRRA mRNA expression in human adipose tissue [43,112]. These initial studies suggest that ESRRA expression may have beneficial effects on the mitochondrial function in muscle and adipose tissue. It is also possible that tissue-specific ESRRA agonists would be helpful in such diseases. Additionally, ESRRA inhibits gluconeogenesis by downregulating *PCK1* and increases FAO via *MCAD* and *CPT1A* upregulation in the liver [1]. Therefore, ESRRA activation may be beneficial in regulating insulin resistance, fatty acid oxidation/oxidative phosphorylation, and hepatic glucose production from gluconeogenesis in diabetic patients.

### 4.4. ESRRA as a Target for Cardiovascular Diseases

ESRRA-PPARGC1A functions as a transcriptional activator in the heart to drive oxidative metabolism. It is also required for the adaptive metabolic and energetic changes that occur during cardiac pressure overload [113,114]. In the hearts of ESRRA null mice, there is a decreased expression of mitochondrial genes involved in energy production, which leads to an impaired mitochondrial function during acute contractile stimulation [115]. Several studies have also suggested that besides ESRRA, ESRRG is also essential for cardiac metabolism and function, and plays an important role in the pathogenesis of cardiovascular diseases, such as cardiac hypertrophy, vascular calcification, heart failure, etc. [113,116,117]. Recently, a natural polyphenolic compound found in red wine, resveratrol, has been shown to improve high-fat diet-induced cardiomyopathy in mice in an ESRRA-dependent manner [118].

## 5. Conclusions

ESRRA has critical roles in mitochondrial homeostasis, energy metabolism, adaptive thermogenesis, and adipogenesis. It enables diverse tissues, such as the liver, BAT, WAT, and muscle, to function during normal physiological conditions, as well as during energy- and nutrient-related stresses. These characteristics make ESRRA an attractive therapeutic target for metabolic disorders. In support of this notion, adipose-specific ESRRA null mice did not show any beneficial effects from adrenergic signaling [22]. ESRRA also plays a critical role in the TH-mediated beta-oxidation of fatty acids, and increases mitochondrial turnover. So far, there have not been any synthetic agonists developed, and the only natural ligand that has been studied to any significant extent has been genistein. Interestingly, whole-body ESRRA knockout in mice offered some protection against DIO, so there may be differences between the whole-body vs. tissue-specific actions of ESRRA Therefore, it is not clear whether ESRRA inhibition or activation will be more beneficial for particular metabolic disorders. It also is possible that ESRRA inhibition by inverse agonists may be beneficial during particular stages of obesity, diabetes, and NAFLD, and agonists useful in others. During aging, ESRRA expression decreases in many tissues, so agonists may be more effective for particular age groups of patients [119,120,121]. Additionally, since ESRRA activity is also regulated by post-translational modifications and co-regulation, combinatorial therapy with drugs that target other mechanisms (e.g., insulin, metformin, etc.) or converging/parallel metabolic pathways may lead to even better outcomes.

## Abbreviations

3MA	Phorbol 12-myristate 13-acetate
BAT	Brown adipose tissue
CR	Calorie restriction
ESRRA/B/G	Estrogen-related receptor alpha/beta/gamma
FAO	Fatty acid beta-oxidation
HFD	High-fat diet
LBD	Ligand binding domain
LOPAC	Library of Pharmacologically Active Compounds
MTOR	Mammalian target of rapamycin
NAFLD	Non-alcoholic fatty liver disease
NASH	Non-alcoholic steatohepatitis
NCOR1	Nuclear receptor co-repressor 1
NR	Nuclear receptor
OXPHOS	Oxidative phosphorylation
PPARGC1A/B	PPAR gamma coactivator 1 alpha/beta
RIP140	Receptor interacting protein 140
TCA	Tricarboxylic acid cycle
TF	Transcription factor
THTHRB	Thyroid hormoneThyroid hormone receptor beta
WAT	White adipose tissue
